# Diurnally dynamic iron allocation promotes N_2_ fixation in marine dominant diazotroph *Trichodesmium*

**DOI:** 10.1016/j.csbj.2023.07.006

**Published:** 2023-07-06

**Authors:** Weicheng Luo, Ya-Wei Luo

**Affiliations:** State Key Laboratory of Marine Environmental Science and College of Ocean and Earth Sciences, Xiamen University, Xiamen 361102, China

**Keywords:** *Trichodesmium*, Dynamic iron allocation, Nitrogen fixation, Respiratory protection, Nitrogenase

## Abstract

*Trichodesmium* is the dominant photoautotrophic dinitrogen (N_2_) fixer (diazotroph) in the ocean. Iron is an important factor limiting growth of marine diazotrophs including *Trichodesmium* mainly because of high iron content of its N_2_-fixing enzyme, nitrogenase. However, it still lacks a quantitative understanding of how dynamic iron allocation among physiological processes acts to regulate growth and N_2_ fixation in *Trichodesmium*. Here, we constructed a model of *Trichodesmium* trichome in which intracellular iron could be dynamically re-allocated in photosystems and nitrogenase during the daytime. The results demonstrate that the dynamic iron allocation enhances modeled N_2_ fixation and growth rates of *Trichodesmium*, especially in iron-limited conditions, albeit having a marginal impact under high iron concentrations. Although the reuse of iron during a day is an apparent cause that dynamic iron allocation can benefit *Trichodesmium* under iron limitation, our model reveals two important mechanisms. First, the release of iron from photosystems downregulates the intracellular oxygen (O_2_) production and reduces the demand of respiratory protection, a process that *Trichodesmium* wastefully respires carbohydrates to create a lower O_2_ window for N_2_ fixation. Hence, more carbohydrates can be used in growth. Second, lower allocation of iron to nitrogenase during early daytime, a period when photosynthesis is active and intracellular O_2_ is high, reduces the amount of iron that is trapped in the inactivated nitrogenase induced by O_2_. This mechanism further increases the iron use efficiency in *Trichodesmium*. Overall, our study provides mechanistic and quantitative insight into the diurnal iron allocation that can alleviate iron limitation to *Trichodesmium*.

## Highlights

• Model of *Trichodesmium* shows diurnally dynamic Fe allocation increases N_2_ fixation.
• Fe allocation increases iron use efficiency in *Trichodesmium.*
• Fe allocation reduces organic carbon wasted in *Trichodesmium* respiratory protection.

## Graphical Abstract

**Figure:**
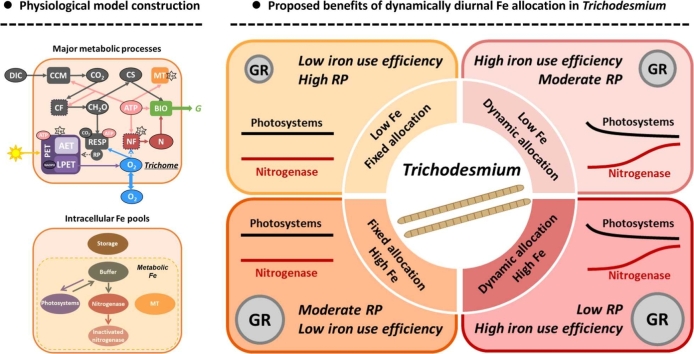

## 1 Introduction

*Trichodesmium* is a dominant cyanobacterial dinitrogen (N_2_)-fixing microorganism and is widely distributed in tropical and subtropical oligotrophic oceans [1]. Iron (Fe) is a key micronutrient needed by *Trichodesmium* mainly for its photosystems and nitrogenase (the enzyme catalyzing N_2_ fixation) [2], [3], [4], [5]. Given that the nitrogenase contains high amount of Fe (2 Fe-protein dimers and 1 Mo­Fe protein per complex) [2] and that the solubility of Fe is low in the ocean [6], Fe is generally a limiting factor for N_2_ fixation and growth of *Trichodesmium* [7], [8], [9].

Many studies have investigated the response of *Trichodesmium* to Fe limitation. *Trichodesmium* generally grows slower and fixes less N_2_ fixation with intensifying Fe limitation, mainly attributed to the downregulation of Fe quota in both photosystems and nitrogenase [8], [10], [11], [12], [13], [14], [15], [16]. Furthermore, some studies have shown that *Trichodesmium* can change the allocation of intracellular Fe in different physiological processes under varying ambient Fe concentration [8], [10], [16]. Under ocean acidification, the low pH decreases the efficiency of nitrogenase, causing the upregulation of nitrogenase under both Fe-depleted and Fe-replete conditions [8], [10], [16].

The majority of these findings of *Trichodesmium* were based on observations without resolving diurnal variations in Fe quota in photosystems and nitrogenase. An exception is the study by [10], showing that the level of photosynthetic proteins decreases and that of nitrogenase increases in *Trichodesmium* cells during the daytime. These results suggest the existence of diurnal reallocation of Fe in photosystems and nitrogenase in *Trichodesmium*, which may increase Fe use efficiency of the organism particularly under Fe limitation.

*Trichodesmium* also faces another challenge that its nitrogenase is irreversibly inactivated by oxygen (O_2_) [17] especially because it performs oxygenic photosynthesis and N_2_ fixation concurrently during the daytime [1]. It has been proposed that *Trichodesmium* can solve the conflict by temporally segregating photosynthesis and N_2_ fixation in different phases and/or spatially segregating them among cells [9], [18], while a recent model study suggests that the spatial segregation may be not necessary [19]. The pattern of diurnal variations in *Trichodesmium* photosystems and nitrogenase mentioned above [10] is consistent with some observed temporal segregations, where photosynthetic and N_2_ fixation rates of *Trichodesmium* were elevated in early and late daytime, respectively [17], [20]. These findings tentatively suggest that diurnal Fe reallocation may also play a role in intracellular O_2_ management in *Trichodesmium*, indirectly regulating photosynthesis and N_2_ fixation.

Nevertheless, it still lacks a systematical understanding of how and to what degree *Trichodesmium* can benefit from the diurnal Fe allocation. A recent model simulated diurnal fluxes of carbon, nitrogen, O_2_, NADPH (nicotinamide adenine dinucleotide phosphate hydrogen) and ATP (adenosine triphosphate) in *Trichodesmium*, but did not simulate its intracellular Fe [19]. In this study, we improved this model by incorporating major intracellular Fe pools including photosystems, nitrogenase, storage and maintenance, so that the diurnal cycles of photosynthesis, N_2_ fixation and O_2_ management could be modulated through the diurnally dynamic allocation of Fe in different pools. The improved model was simulated under both Fe-replete and Fe-depleted conditions. Model experiments were also conducted with diurnally fixed Fe in different pools. The comparison of model results provided us with a mechanistic and quantitative understanding of the role of dynamic Fe allocation in *Trichodesmium*. This framework is expected to improve the projections of N_2_ fixation by this dominant diazotroph in the ocean with variations in Fe availability over space and time.

## 2 Methods

The model in this study was constructed by modifying a previous model [19] with new representations of intracellular Fe pools and Fe-related processes. In the following, we briefly describe the model schemes focusing on the new schemes, while the full model description, parameter values and variables can be found in Supplementary Methods and Tables S1 to S4.

### 2.1 General model framework

**Fig. 1:**
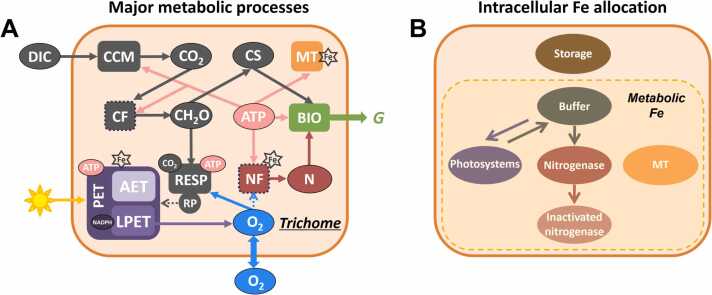
Model structure of the physiological model with intracellular Fe allocation in *Trichodesmium*. (A) Photosynthesis, N_2_ fixation and other major processes simulated in *Trichodesmium* trichome. To simplify the plot, arrows that represent the ATP production by PET and ordinary respiration, the production NADPH by LPET, and the NADPH consumption by carbon and N_2_ fixations (dotted frames) are omitted. ATP produced by RP is wasted as heat and not counted. The pentagrams with Fe indicate processes requiring Fe. Dark orange frame, cell membrane; oval blobs, biochemical pools; rectangles, metabolic processes; solid arrows, mass or energy fluxes; dashed arrows, inhibition effects of RP on PET (gray) or O_2_ on N_2_ fixation (blue). LPET: linear photosynthetic electron transfer; AET: alternative electron transfer; NADPH: nicotinamide adenine dinucleotide phosphate hydrogen; ATP: adenosine triphosphate; DIC: dissolved inorganic carbon; CCM: CO_2_ concentrating mechanism; CF: carbon fixation; NF: N_2_ fixation; RP: respiratory protection; RESP: ordinary respiration; CH_2_O: carbohydrate; CS: carbon skeleton; N: fixed nitrogen; MT: maintenance; BIO: biosynthesis; *G*: growth rate. (B) The model represents fundamental intracellular Fe pools, including storage and five metabolic pools (photosystems, active and inactivated nitrogenase, maintenance, buffer and storage). Note that all inactivated nitrogenase by O_2_ does not decompose and Fe in it is not recycled during model period (daytime).

The model (Fig. 1) simulates diurnal cycles of carbon assimilation into carbon skeleton and carbohydrate and N_2_ fixation in *Trichodesmium* trichome. The rates of these processes are controlled by dynamic allocations of Fe, ATP and NADPH across different metabolic processes and by the regulation of intracellular O_2_. The model simulates daily growth rate of *Trichodesmium* as a function of dissolved inorganic Fe concentration (Fe′) in the extracellular environment. The model runs for 12 h light period, while biosynthesis is designed to occur during the dark period and the temporal variation of the biosynthesis is not simulated [20]. Instead, the accumulated carbon skeletons, carbohydrates and fixed N at the end of the light period are used to calculate the synthesized biomass and the growth rate. The model parameters are optimized to maximize the growth rate.

Photosynthetic electron transfer (PET) includes linear PET (LPET) and alternative electron transfer (AET) in our model. Both LPET and AET produce ATP, while NADPH required by carbon and N_2_ fixations is only produced by LPET [21], [22]. ATP is consumed by carbon and N_2_ fixations, CCM, maintenance, and biosynthesis. N_2_ fixation can occur only when the intracellular O_2_ concentration is low. The O_2_ produced by LPET [21], [22] can physically diffuse between cells and the extracellular environment. O_2_ can be also consumed by respiratory protection, a process that actively respires carbohydrates to consume intracellular O_2_ and protect N_2_ fixation, with the produced energy lost in the form of heat to the environment [17], [19], [23], [24], [25].

The model calculates the total intracellular Fe from Fe′ using a previous model scheme [16] based on observations [10]. A portion of intracellular Fe is stored and does not involve in metabolic processes, while a small, fixed portion of metabolic Fe is used in maintenance (Fig. 1B). The remaining Fe is dynamically allocated among photosystems, active and inactivated nitrogenase, and buffer during the daytime (Fig. 1B). Active nitrogenase is inactivated by intracellular O_2_ [17]. The decomposition of nitrogenase seems to occur at night [26] and therefore is not considered during the light period in our model. That is, the Fe in the inactivated nitrogenase is not reused in the light period. This model case with diurnally dynamic Fe allocation is referred to as the “dynamic-Fe” case hereafter.

To further quantitatively explore the role of dynamically diurnal Fe allocation, we set up another model case with fixed Fe allocation (referred to as “fixed-Fe” case), in which the Fe in photosystems, nitrogenase and buffer are constant during the modeled light period, except for the O_2_-induced inactivation of the active nitrogenase.

Both model cases were run under a Fe-depleted condition (Fe′ = 40 pM) and a Fe-replete condition (Fe′ = 1250 pM).

### 2.2 Photosynthetic pathways

The total PET rate [$V_{\mathrm{PET}}$, mol electron (mol C)^-1^ s^-1^] is regulated by light intensity (I, μmol m^-2^ s^-1^) and the Fe quota in photosystems [${\mathrm{Fe}}_{\mathrm{PS}}$, μmol Fe (mol C)^-1^]. $V_{\mathrm{PET}}$ is inhibited by respiratory protection (RP) [$V_{\mathrm{RP}}$, mol C (mol C)^-1^ s^-1^, described later] [17].

(1) $$\begin{array}{c} {\text{V}}_{\text{PET}}\;=\;{\text{v}}_{\text{PET}}^{max}\text{·}\frac{{\text{Fe}}_{\text{PS}}}{{\text{Fe}}_{\text{PS}}\;+\;{\text{k}}_{\text{Fe}}^{\text{PS}}}\text{·}\\ \;\;\;\;\;\;\;\;\;\;\;\;\;\;(1\;-\;{\text{e}}^{\;-\;\alpha_{\text{I}}\text{·I}}){\text{·e}}^{\;-\;{\text{β·V}}_{\text{RP}}} \end{array}$$

where $v_{\mathrm{PET}}^{\max}$ [mol electron (mol C)^-1^ s^-1^] is the maximal rate of PET, $k_{\mathrm{Fe}}^{\mathrm{PS}}$ [μmol Fe (mol C)^-1^] is the half-saturating coefficient of ${\mathrm{Fe}}_{\mathrm{PS}}$ for PET, $\alpha_{I}$ (μmol^-1^ m^2^ s) is the initial slope of PET versus light curve, and β [mol C (mol C)^-1^ s] represents the degree of the inhibition from RP on PET.

The PET electrons are further differentiated into two pathways: LPET and AET. The fraction of electrons flowing into LPET and AET is calculated at each time step to fulfill the immediate intracellular demands for ATP and NADPH, as LPET produces both ATP and NADPH while AET only produces ATP [19].

### 2.3 N_2_ fixation

N_2_ fixation requires both ATP and NADPH [27], [28]. The maximal potential of N_2_ fixation rate [$V_{\mathrm{NF}}^{\max}$, mol N (mol C)^-1^ s^-1^] is determined when the produced ATP and NADPH from PET are completely consumed by N_2_ fixation [19].

The N_2_ fixation rate [$V_{\mathrm{NF}}$, mol N (mol C)^-1^ s^-1^] is also limited by the Fe quota in nitrogenase [${\mathrm{Fe}}_{\mathrm{NF}}$, μmol Fe (mol C)^-1^] and inhibited by intracellular O_2_ [$O_{2}$, mol O_2_ m^-3^] [29].

(2) $${\text{V}}_{\text{NF}}\;=\;{\text{V}}_{\text{NF}}^{\max}\text{·}\frac{{\text{Fe}}_{\text{NF}}}{{\text{Fe}}_{\text{NF}}\;+\;{\text{k}}_{\text{Fe}}^{\text{NF}}}\text{·}(1\;-\;\frac{{\text{O}}_{2}}{{\text{O}}_{2}\;+\;{\text{k}}_{{\text{O}}_{2}}^{\text{NF}}\;})$$

where $k_{\mathrm{Fe}}^{\mathrm{NF}}$ [μmol Fe (mol C)^-1^] and $k_{O_{2}}^{\mathrm{NF}}$ (mol O_2_ m^-3^) are half-saturating coefficients of ${\mathrm{Fe}}_{\mathrm{NF}}$ and $O_{2}$ for N_2_ fixation.

### 2.4 Carbon fixation

Carbon fixation also requires both NADPH and ATP [30]. The carbon fixation rate is solved at each time step assuming that total NADPH and ATP production by PET are immediately and fully utilized by intracellular process [19]. Carbohydrates, produced by carbon fixation, stimulate the production of carbon skeletons which are downregulated by its own accumulation.

### 2.5 Respiratory protection and O_2_ diffusion

RP is a process wastefully respiring carbohydrates in order to reduce intracellular O_2_ for supporting N_2_ fixation. The RP rate increases with the requirement for N_2_ fixation but decreases as intracellular O_2_ level rises [19].

The rate of O_2_ diffusion between intracellular cytoplasm and ambient environment is parameterized by adopting the scheme of [31].

### 2.6 Intracellular Fe pools and translocation

*Trichodesmium* can take up more Fe than that required for its metabolism (called ‘luxury uptake’) especially in high-Fe environments, and the excess Fe is stored for surviving in low-Fe environments [32], [33]. The fractions of total intracellular Fe used in metabolism and storage are calculated using a previous scheme [16].

Fe in maintenance accounts for 10% of metabolic Fe. Fe used in the photosystems [${\mathrm{Fe}}_{\mathrm{PS}}$, μmol Fe (mol C)^-1^] and nitrogenase [${\mathrm{Fe}}_{\mathrm{NF}}$, μmol Fe (mol C)^-1^] is from the buffer pool [${\mathrm{Fe}}_{\mathrm{BF}}$, μmol Fe (mol C)^-1^] (Fig. 1B). The synthesis rate of photosystems [$T_{\mathrm{PS}}^{\mathrm{BF}}$, μmol Fe (mol C)^-1^ s^-1^] is stimulated by light intensity and is gradually saturated with ${\mathrm{Fe}}_{\mathrm{PS}}$:

(3) $$\begin{array}{c} {\text{T}}_{\text{PS}}^{\text{BF}}\;=\;{\text{T}}_{{\text{PS}}_{\max}}^{\text{BF}}\text{·}(1\;-{\text{e}}^{\;-\;\alpha_{\text{I}}\text{·I}})\text{·}\\ \;\;\;\;\;\;\;\;\;\;\;\;\;\;\;\;\;\;\;\;\;\;\;\;(1\;-\;\frac{{\text{Fe}}_{\text{PS}}}{{\text{Fe}}_{\text{PS}}+\;{\text{k}}_{{\text{Fe}}_{\text{PS}}}^{{\text{PS}}_{\text{syn}}}}) \end{array}$$

where $T_{{\mathrm{PS}}_{\max}}^{\mathrm{BF}}$ [μmol Fe (mol C)^-1^ s^-1^] is the maximal synthesis rate of photosystems, $k_{{\mathrm{Fe}}_{\mathrm{PS}}}^{{\mathrm{PS}}_{\mathrm{syn}}}$ [μmol Fe (mol C)^-1^] is a half-saturating coefficient of ${\mathrm{Fe}}_{\mathrm{PS}}$.

The proteins in photosystems decompose and release Fe back into the buffer pool. The decomposition rate of photosystems [$T_{\mathrm{BF}}^{\mathrm{PS}}$, μmol Fe (mol C)^-1^ s^-1^] is assumed to be stimulated by ${\mathrm{Fe}}_{\mathrm{PS}}$ but inhibited by respiratory protection [17]:

(4) $${\text{T}}_{\text{BF}}^{\text{PS}}\;=\;{\text{T}}_{{\text{BF}}_{\max}}^{\text{PS}}\text{·}\frac{{\text{Fe}}_{\text{PS}}}{{\text{Fe}}_{\text{PS}}\;+\;{\text{k}}_{{\text{Fe}}_{\text{PS}}}^{{\text{PS}}_{\text{dec}}}}\text{·}{\text{e}}^{\;-\;\text{β·}{\text{V}}_{\text{RP}}}$$

where $T_{{\mathrm{BF}}_{\max}}^{\mathrm{PS}}$ [μmol Fe (mol C)^-1^ s^-1^] is the maximal decomposition rate of photosystems, $k_{{\mathrm{Fe}}_{\mathrm{PS}}}^{{\mathrm{PS}}_{\mathrm{dec}}}$ [μmol Fe (mol C)^-1^] is the half-saturating coefficient of ${\mathrm{Fe}}_{\mathrm{PS}}$ for the decomposition of photosystems.

The synthesis rate of active nitrogenase from the buffer pool [$T_{\mathrm{NF}}^{\mathrm{BF}}$, μmol Fe (mol C)^-1^ s^-1^] increases with intracellular requirement for N_2_ fixation (Φ) and ${\mathrm{Fe}}_{\mathrm{BF}}$:

(5) $$\text{Φ}\;=\;(1\;-\;{\text{e}}^{\;-\;\alpha_{\text{I}}\text{·I}})\text{·}\frac{\text{CS}}{\text{CS}\;+\;{\text{k}}_{\text{CS}}}\text{·}(\frac{{\text{N}}_{\max}\;-\text{N}}{{\text{N}}_{\max}})$$

(6) $${\text{T}}_{\text{NF}}^{\text{BF}}\;=\;{\text{T}}_{{\text{NF}}_{\max}}^{\text{BF}}\text{·}\text{Φ}\text{·}\frac{{\text{Fe}}_{\text{BF}}}{{\text{Fe}}_{\text{BF}}\;+\;{\text{k}}_{{\text{Fe}}_{\text{BF}}}^{{\text{NF}}_{\text{syn}}}}$$

where $T_{{\mathrm{NF}}_{\max}}^{\mathrm{BF}}$ [μmol Fe (mol C)^-1^ s^-1^] is the maximal nitrogenase synthesis rate, $k_{\mathrm{CS}}$ [mol C (mol C)^-1^] and $k_{{\mathrm{Fe}}_{\mathrm{BF}}}^{{\mathrm{NF}}_{\mathrm{syn}}}$ [μmol Fe (mol C)^-1^] are half-saturating coefficients of the carbon skeleton and ${\mathrm{Fe}}_{\mathrm{BF}}$ for the synthesis of nitrogenase, respectively; $N_{\max}$ [mol N (mol C)^-1^] is the maximal N storage. Hence, this scheme assumes that the increase of light and the production of CS stimulates the requirement of N_2_ fixation while the accumulation of fixed N lowers the requirement.

Active nitrogenase is inhibited upon exposure to O_2_, flowing into the pool of inactivated nitrogenase [17] at the rate [μmol Fe (mol C)^-1^ s^-1^]:

(7) $${\text{T}}_{\text{NF}}^{\text{NA}}\;=\;{\text{T}}_{{\text{NF}}_{\max}}^{\text{NA}}\text{·}\frac{{\text{Fe}}_{\text{NF}}}{{\text{Fe}}_{\text{NF}}\;+\;{\text{k}}_{\text{Fe}}^{\text{NF}}}\text{·}\frac{{\text{O}}_{2}}{{\text{O}}_{2}\;+\;{\text{k}}_{{\text{O}}_{2}}^{\text{NF}}}$$

where $T_{{\mathrm{NF}}_{\max}}^{\mathrm{NA}}$ [μmol Fe (mol C)^-1^ s^-1^] is the maximal inactivation rate of nitrogenase. It should be noted that in the fixed-Fe model case, photosystems and total nitrogenase are set diurnally constant, but the inactivation of nitrogenase still occurs. The Fe in the inactivated nitrogenase is not released for reuse during the model period, as discussed above.

### 2.7 Model parameter values

Some model parameters were optimized to maximize *Trichodesmium* growth rate (Table S1). In the fixed-Fe case, three parameters, including the maximal respiratory protection rate ($v_{\mathrm{RP}}^{\max}$), the initial fraction of metabolic Fe in photosystems ($f_{{\mathrm{Fe}}_{0}}^{\mathrm{PS}}$), and the initial fraction of metabolic Fe in nitrogenase ($f_{Fe_{0}}^{NF}$), were optimized (Table S1). In the dynamic-Fe case, $f_{{\mathrm{Fe}}_{0}}^{\mathrm{PS}}$ and $f_{Fe_{0}}^{NF}$ were not optimized but were set using observations in a laboratory culture study [10]. In addition to $v_{\mathrm{RP}}^{\max}$, the maximal synthesis ($T_{{\mathrm{PS}}_{\max}}^{\mathrm{BF}}$) and decomposition ($T_{{\mathrm{BF}}_{\max}}^{\mathrm{PS}}$) rates of photosystems and the maximal synthesis rate ($T_{{\mathrm{NF}}_{\max}}^{\mathrm{BF}}$) of nitrogenase were also optimized (Table S1). The optimization was conducted using the global optimizer MultiStart in MATLAB.

Other parameters that were not optimized (Table S2) were either adopted from previous studies or derived from our constant-light model experiments that fit the observed growth and N_2_ fixation rates and the observed diurnal Fe in photosystems and nitrogenase [10] (*see* Results). The Fe in nitrogenase and photosystems from [10] were estimated from observed protein content, based on Fe atoms in per protein. PSII, Cyt *b6f*, PSI and Ferredoxin together represents photosystems. Cyt *b6f* and Ferredoxin were not measured in [10] but estimated by assuming Cyt *b6f*:PSII = 1:1 in Fe quota and Ferredoxin:PSI = 1:1 in protein content. Further details are in the supplementary information in [16].

## 3 Results

### 3.1 Simulated growth rate, carbon and N_2_ fixation rates and O_2_ concentration

**Fig. 2:**
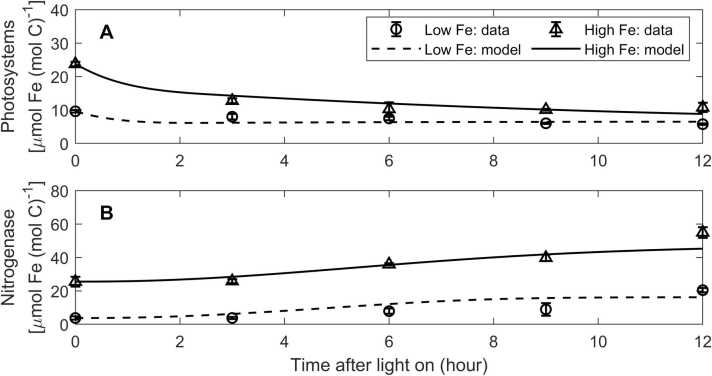
Modeled and observed diurnal variations of Fe in photosystems and nitrogenase. The observational data are from [10]. The model is simulated with dynamic Fe allocation under both low-Fe and high-Fe conditions. Light intensity in the model is diurnally constant as that in [10]. Error bars represent one standard deviation.

We first used the observational data from a culture experiment [10] to constrain and evaluate the model results. Because the light intensity was constant in this culture experiment, we used constant light intensity (90 μmol m^-2^ s^-1^) and dynamic-Fe case in this model exercise, so that the model results and the observational data can be compared. By tuning values of some model parameters (Table S2), the model results well fitted the observed daily growth rates (Table 1). Additionally, the model also captured the diurnal variations in the observed photosystem and nitrogenase Fe pools (for photosystem Fe, *R*^*2*^ = 0.47 and 0.93 under low and high Fe, respectively; for nitrogenase Fe, *R*^*2*^ = 0.53 and 0.82 under low and high Fe, respectively) (Fig. 2). The observations and the model both showed that nitrogenase increased and photosystem proteins decreased over the day period under the low-Fe and high-Fe conditions. These results partly support the robustness of our model. We used these tuned model parameters in our following simulations, with the exception of those key model parameters optimized for maximal growth rates (*see* Section 2.7 and Table S1).

**Table 1:** Comparison of modeled and observed growth rates under diurnally constant light intensity.

	Low Fe (40 pM)	High Fe (1250 pM)
Observed growth rate (d^-1^)*	0.26 ± 0.02	0.46 ± 0.01
Modeled Growth rate (d^-1^)	0.26	0.47
Model-observation comparison (*p* of z-test)	0.99	0.57

* Data are from [10], mean ± standard deviation.

**Fig. 3:**
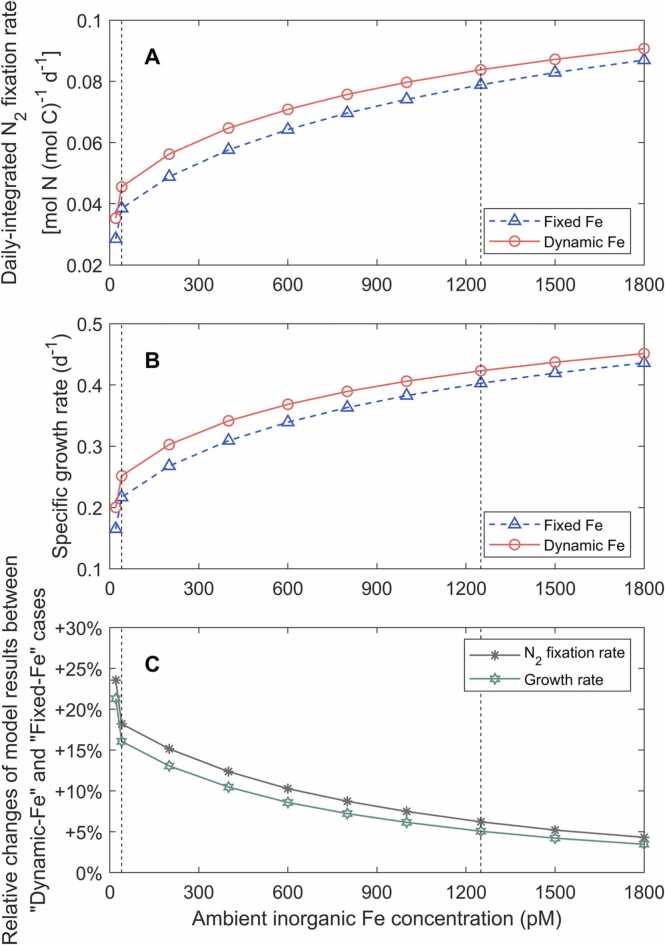
Simulated daily-integrated N_2_ fixation rates (A), growth rates (B) and their relative changes (C) between “Dynamic-Fe” and “Fixed-Fe” cases under different ambient inorganic iron concentrations. Black dashed lines represent ambient inorganic iron concentrations (40 and 1250 pM) used in the standard model cases.

We then ran the model with diurnally dynamic light intensity using the sine function during a 12-hour light period [6] to simulate more natural conditions. The simulations were extended to ten Fe levels ranging from 20 pM to 1800 pM. The model results showed that N_2_ fixation and growth rates of *Trichodesmium* increased with the increase of the Fe concentration (Fig. 3), which generally captured the trends of observed N_2_ fixation and growth rates in previous culturing experiments [8]. Furthermore, with increasing Fe concentration, the impact of dynamic Fe allocation in promoting growth rates decreased from 21% to 3% (Fig. 3), highlighting that the benefit of the dynamic Fe allocation tended to be more pronounced under low Fe and could be marginal under high Fe.

In the following, we analyzed the effect of dynamic Fe allocation by comparing the dynamic-Fe and fixed-Fe model cases using diurnally dynamic light intensity. We focused on the simulation results at two Fe levels (40 pM and 1250 pM) that were used in the laboratory experiments [10]. Although the growth rates were higher in the dynamic-Fe cases than in the fixed-Fe cases, the gross carbon fixation rates in the former were even lower under both low- and high-Fe conditions (Table 2). In other words, the dynamic Fe allocation improved the carbon use efficiency (ratio of net to gross carbon production) (Table 2). Additionally, the simulated higher carbon use efficiency under higher Fe was also consistent with previous studies [13], [34].

**Table 2:** Modeled daily-integrated rates of *Trichodesmium*.

Model cases	Growth rate	Gross carbon fixation rate	N_2_ fixation rate	Gross fixed C:N	Gross fixed carbon used by respiratory protection	Carbon use efficiency*
	(d^-1^)	[mol C (mol C)^-1^ d^-1^]	[mol N (mol C)^-1^ d^-1^]	[mol C (mol N)^-1^]		
	Low Fe (40 pM)					
Fixed-Fe	0.22	3.1	0.039	81	89%	8%
Dynamic-Fe	0.25	2.9	0.045	64	86%	10%
	High Fe (1250 pM)					
Fixed-Fe	0.40	3.8	0.079	48	81%	13%
Dynamic-Fe	0.42	3.6	0.084	43	79%	15%

* Carbon use efficiency: percentage of gross carbon production assimilated to biomass.

**Fig. 4:**
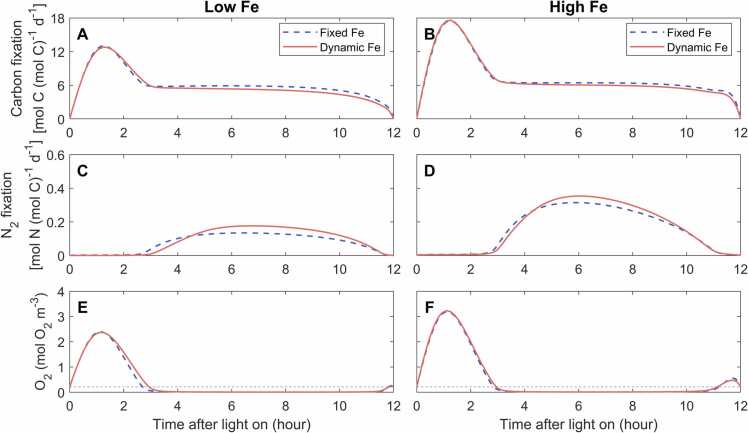
Simulated instantaneous rates of gross carbon fixation and N_2_ fixation and intracellular O_2_ concentrations during the light period. The model is simulated with diurnally fixed and dynamic Fe allocations under low-Fe (40 pM) (A, C and E) and high-Fe (1250 pM) (B, D and F) conditions.

The modeled carbon and N_2_ fixation rates exhibited diurnal variations (Fig. 4). Under low Fe, carbon fixation in the two model cases (Fig. 4A) increased and reached a maximal level within the first 1.5 h, decreased by approximately 50% in the next 1.5 h, maintained nearly unchanged for another 6 h, and then decreased again until it ceased at the end of the daytime. N_2_ fixation mainly occurred during the midday and late light period (Fig. 4C) when carbon fixation was downregulated (Fig. 4A) and intracellular O_2_ was low (Fig. 4E). Compared to the fixed-Fe case, the carbon fixation rate during the low-O_2_ window period was slightly lower in the dynamic-Fe case (Fig. 4A, E); the N_2_ fixation rate in the dynamic-Fe case peaked later but at a higher level and thus achieved a higher daily-integrated rate (Fig. 4C and Table 2). Under high Fe, modeled carbon and N_2_ fixation rates were higher than those under low Fe, while their diurnal patterns were similar under both Fe levels (Fig. 4A–D).

### 3.2 Simulated diurnal Fe allocation

**Fig. 5:**
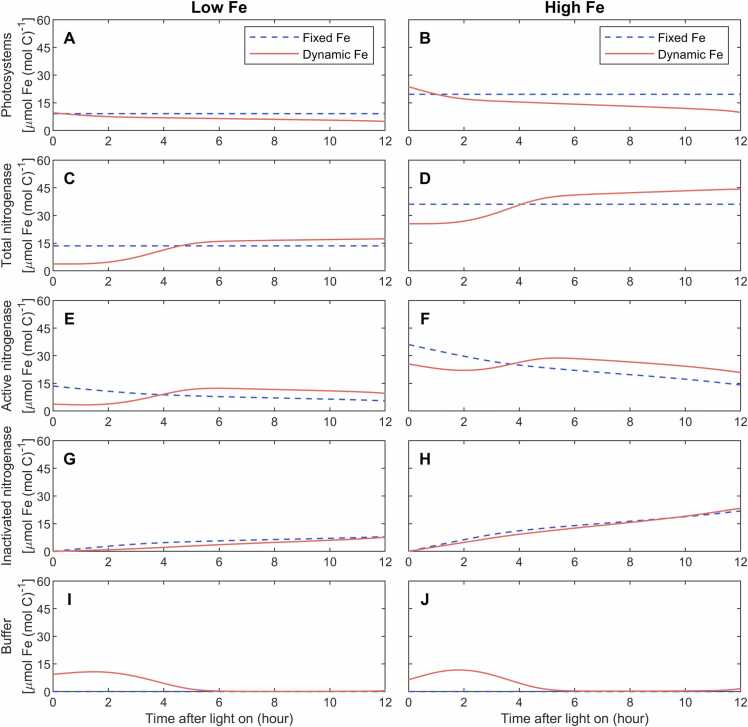
Simulated diurnal variations of Fe in photosystems, total, active and inactivated nitrogenase, and buffer. The model is simulated with diurnally fixed or dynamic Fe allocations under low-Fe (40 pM) (A, C, E, G and I) and high-Fe (1250 pM) (B, D, F, H and J) conditions.

In the dynamic-Fe case, initial levels of Fe in photosystems and nitrogenase at the beginning of the light period were set based on observations in [10] under both low and high Fe. Fe in photosystems decreased in the whole daytime, with the decrease rate being slowed after 3 h (Fig. 5A, B). This decomposition of photosystem Fe can then supplement the buffer pool in the dynamic-Fe case (Fig. 5I, J). Active nitrogenase was continuously inactivated during the whole light period in both model cases, while the inactivation was slower in middle and late daytime when intracellular O_2_ was low (Fig. 5G, H). Consequently, active nitrogenase decreased over time in the fixed-Fe case (Fig. 5E, F). In the dynamic-Fe case, however, although initial nitrogenase was less than that in the fixed-Fe case, Fe from buffer pools could support continuous synthesis of new nitrogenase (Fig. 5C, D) and the active nitrogenase became more than that in the fixed-Fe case during the period of active N_2_ fixation (3 −7 h) (Fig. 5E, F). It was worth noting that in the dynamic-Fe case, because of the lower level of nitrogenase in the early light period (Fig. 5C, D), less Fe was trapped in the inactivated nitrogenase in most time during the day (Fig. 5G, H), promoting overall Fe use efficiency in the model.

This diurnal pattern of Fe pools largely determined the modeled photosynthesis and N_2_ fixation. During the early daytime (0 −3 h), large and close amount of Fe in photosystems in the fixed-Fe and dynamic-Fe cases (Fig. 5A, B) ensured high and similar photosynthesis rates (Fig. 4A, B). After 3 h, the reduced Fe in photosystems in the dynamic-Fe case (Fig. 5A, B) resulted in a lower photosynthesis rate than that in the fixed-Fe case (Fig. 4A, B). In the dynamic-Fe case, nitrogenase can start at a very low level because of unfavorable condition for N_2_ fixation in the morning (0 −3 h) (Fig. 4E, F; 5 C, D); in approximately 3 −4 h, higher levels of active nitrogenase supported higher N_2_ fixation rate in the fixed-Fe case (Fig. 4C, D; 5E, F). However, with the synthesis of nitrogenase in the dynamic-Fe case (Fig. 5C, D), its active nitrogenase exceeded that in the fixed-Fe case after 4 h (Fig. 5E, F), achieving a higher N_2_ fixation rate (Fig. 4C, D). The accumulated fixed N in the dynamic-Fe then became more than that in the fixed-Fe case after 7 h (Fig. S1E).

Comparing the low-Fe and the high-Fe simulations, the Fe allocations presented similar patterns, except for higher magnitudes of variations in the high-Fe simulation (Fig. 5). In the dynamic-Fe case, a higher fraction of metabolic Fe was allocated to initial nitrogenase under high Fe than under low Fe (41% versus 15%) (Fig. 5D). Consequently, the relative difference in inactivated nitrogenase between the fixed-Fe and dynamic-Fe cases was smaller under high Fe than under low Fe (Fig. 5G, H), which weakens the potential role of dynamic allocation in promoting N_2_ fixation and growth rates (Table 2).

## 4 Discussion

In this study, we constructed an eco-physiological model of *Trichodesmium* trichome to quantitatively study how its intracellular Fe was diurnally allocated to photosynthetic and N_2_-fixing apparatuses under Fe limitation and repletion during the light period (Fig. 1). The model also integrated intracellular management including the formation of the temporal segregation between photosynthesis and N_2_ fixation and the creation of the low-O_2_ window (Fig. 4). The model results reproduced the observed diurnal variations of Fe in photosystems and nitrogenase of *Trichodesmium* [10], showing that photosystems decrease while nitrogenase increases continuously during the daytime (Fig. 2, Fig. 5). It also captured the general diurnal patterns of carbon and N_2_ fixations found in some observations of *Trichodesmium* [17], [20]: high photosynthesis and low N_2_ fixation in the morning, and moderate photosynthesis and high N_2_ fixation in the noon and afternoon. The model results showed that the dynamic Fe allocation moderately increased the *Trichodesmium* growth rates (Table 2).

We also designed a new model experiment using the dynamic-Fe case but optimizing the initial Fe in photosystems and nitrogenase for maximal growth rate, instead of setting the initial Fe to observed values. Compared to the standard dynamic-Fe case, this setup further increased the growth rate by 18% and 10% under the low and high Fe, respectively. In other words, if the initial Fe pools could be more flexible, the dynamic Fe allocation would generate even higher potential of benefit for *Trichodesmium* (Figs. S2−4).

### 4.1 Dynamic Fe allocation decreases the requirement of respiratory protection

**Fig. 6:**
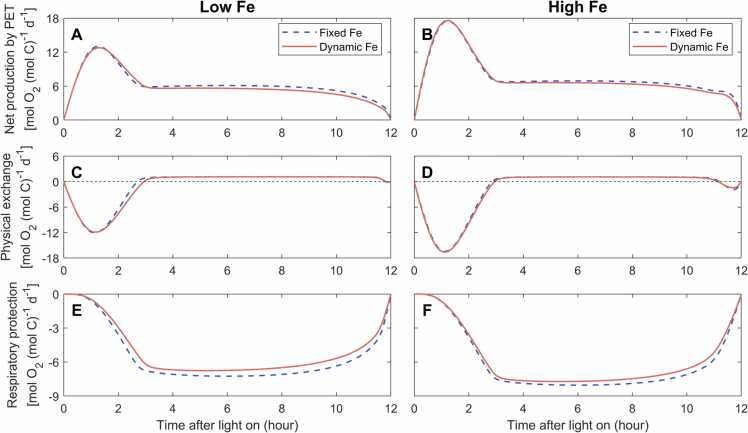
Simulated diurnal intracellular O_2_ fluxes. The model is simulated with diurnally fixed or dynamic Fe allocations under low-Fe (40 pM) (A, C and E) and high-Fe (1250 pM) (B, D and F) conditions. Positive physical O_2_ exchange represents O_2_ flux into cells. Negative respiratory protection represents O_2_ consumption.

In the dynamic-Fe case, reduced Fe in photosystems after 3 h (Fig. 5A) downregulated the rate of O_2_ production by 6% and 3% compared to the fixed-Fe case under low Fe and high Fe, respectively (Fig. 6A, B). Hence, the respiratory protection needed to create a low O_2_ window for N_2_ fixation decreased by 9% and 5% (Fig. 6E, F). Respiratory protection has been proposed as an important intracellular O_2_ management strategy in *Trichodesmium* and other marine non-heterocystous diazotrophs [17], [19], [35], [36], [37]. However, respiratory protection is generally a high indirect cost for N_2_ fixation, as evidenced by observed high daily-integrated gross fixed C:N ratios (e.g., 30 – 50 under high Fe) [17], [20], [38], [39]. The process consumes a large fraction of gross produced carbohydrate (Table 2) without supplying energy for metabolic processes [17], [23], [24], [25]. This indicates that dynamic Fe allocation might play a potential role in improving the carbon use efficiency of *Trichodesmium* by downregulating photosystems and photosynthetic O_2_ production during the period of active N_2_ fixation (Table 2).

### 4.2 Dynamic Fe allocation promotes Fe use efficiency

Our model results revealed a mechanism that dynamic Fe allocation promoted Fe use efficiency for N_2_ fixation and growth in *Trichodesmium*. The dynamic Fe allocation allowed for relatively low Fe allocation to nitrogenase during the early daytime (Fig. 5C, D) when photosynthesis was active (Fig. 4), so that less Fe was trapped in the nitrogenase inactivated by high intracellular O_2_ during this period (Fig. 5G, H).

In our further model experiment in which the inactivated nitrogenase was instantaneously decomposed and its Fe returned to the buffer pool immediately, the dynamic-Fe case then did not simulate higher growth rates than the fixed-Fe case. However, the results of this model experiment were related to the initial Fe of photosystems and nitrogenase using the results from a previous culture experiment [10] (Figs. S2−4). When the initial Fe in photosystems was maximized to 90% of total metabolic Fe (the rest 10% Fe was used in maintenance) and the instant decomposition of inactivated nitrogenase was implemented, the dynamic-Fe case could still generate substantially higher growth rates (9% and 4% under low Fe and high Fe, respectively) than the fixed-Fe case. That was mainly because the higher initial content of photosystems promoted carbon fixation and more carbohydrates were accumulated in early daytime (0 −2 h), and the large amount of Fe in photosystems was then released to support the synthesis of nitrogenase after this period (Fig. S5). Nevertheless, a previous study showed that the decomposition of nitrogenase did not occur during the light period [26]. The benefits from the dynamic Fe allocation shown in the standard model cases likely occurs in *Trichodesmium*. Meanwhile, the timescales of the recovery of nitrogenase by de novo synthesis need to be further explored to better evaluate the role of dynamic Fe allocation in *Trichodesmium*.

**Fig. 7:**
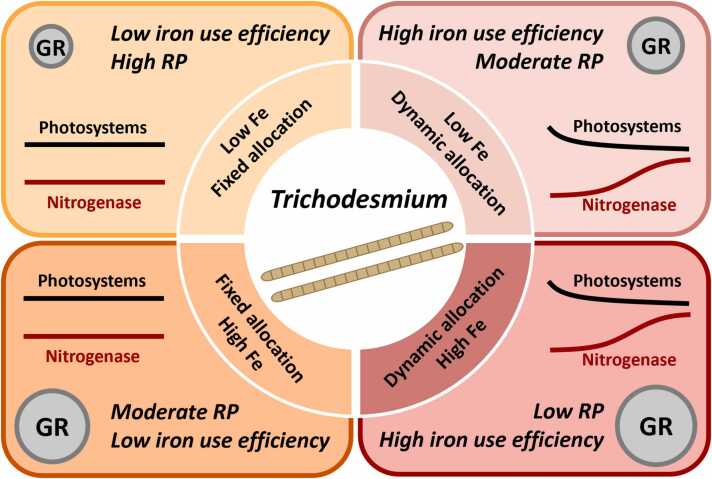
Schematic diagram illustrating diurnally fixed and dynamic Fe allocations under low-Fe and high-Fe conditions. Sizes of bubble charts represents modeled growth rates (GR). Dynamic Fe allocation can reduce the requirement of respiratory protection (RP), calculated as the ratio of daily-integrated carbon consumption rate by respiratory protection to daily-integrated gross carbon fixation rate. It can also improve the iron use efficiency based on the ratio of growth rate to intracellular Fe.

In summary of these analyses, our model results suggest that diurnally dynamic Fe allocation can improve carbon and Fe use efficiency, as well as the growth rate of *Trichodesmium* particularly under low Fe, thereby partly alleviating Fe limitation (Fig. 7). Our model study highlights two main potential mechanisms explaining the benefits of dynamic Fe allocation to N_2_ fixation and growth in *Trichodesmium*.

Note that the Fe in respiratory electron transfer chain was not simulated due to lacking diurnal observational data, although it might occupy a small fraction of intracellular Fe pool (less than 10% of the total intracellular Fe) in *Trichodesmium* [40]. In fact, respiratory and photosynthetic electron transfer chains share several apparatuses (e.g., Cyt *b6f* complex and ferredoxin) in *Trichodesmium* [11], suggesting that Fe used only by respiratory processes could be even less.

### 4.3 Broader context: Is dynamic Fe allocation a common strategy in marine cyanobacterial diazotrophs?

Although the pattern of diurnally dynamic Fe allocation in *Trichodesmium* is not exactly the same as those in other marine cyanobacterial diazotrophs, several potential benefits of dynamic Fe allocation revealed by our model can be common in certain marine diazotrophs (Table 3).

**Table 3:** The list of dynamic Fe allocation with potential benefits in some marine autotrophic diazotrophs.

Diazotroph	Dynamic Fe allocation	Ref.
*Trichodesmium*	Yes	[10]
UCYN-A	Possible	[41]
UCYN-B (*Crocosphaera*)	Yes	[37], [44]
UCYN-C	Yes	[41], [43]
Diazotroph-diatom association (DDA)	No	[42]

The unicellular N_2_-fixing cyanobacterial group A (UCYN-A) symbioses with haptophyte algae and lacks O_2_-envolving photosystem II [9]. Similar to *Trichodesmium*, UCYN-A symbiosis conducts photosynthesis and N_2_ fixation during daytime, while its diurnal transcriptomic patterns of genes relevant to photosystem I and nitrogenase also segregate [41]. High transcription of photosystem I in UCYN-A during the early light period may contribute to reducing intracellular O_2_ by cyclic photosynthetic electron transfer; and its decreasing transcription level indicates Fe release from photosystems, which can be a stage preparing for high transcription of nitrogenase and high N_2_ fixation rate later [41]. This suggests a possibility that UCYN-A can also dynamically allocate intracellular Fe between photosystem I and nitrogenase to improve the Fe use efficiency, and possibly to also lower respiratory protection as revealed by rare cytochrome *c* oxidase *coxA* gene transcription in UCYN-A during the daytime [23], [41]. For diazotroph-diatom associations (DDAs) that form heterocyst to protect nitrogenase, there is no obvious dynamic Fe allocation between photosystems and nitrogenase [42]. This suggests that the dynamic allocation of Fe may not be necessary when photosynthesis and N_2_ fixation are physically segregated.

Unlike *Trichodesmium* and UCYN-A, *Crocosphaera* (UCYN-B) performs photosynthesis in the day but conducts N_2_ fixation at night [41], [43], [44]. During the light period, nearly no Fe in *Crocosphaera* is allocated to nitrogenase, while most of its intracellular Fe is in photosystems [37], [44]. This strategy helps prevent the inactivation of nitrogenase during the light period when photosynthetic O_2_ production is active and intracellular O_2_ concentration is high [37]. During the dark period, the majority of intracellular Fe is released from photosystems and used to synthesize nitrogenase [37], [44]. Additionally, respiratory protection at low levels is sufficient to protect nitrogenase and support N_2_ fixation [35], [37]. This dynamic allocation of Fe in the light and dark periods may also partly explain why *Crocosphaera* is more abundant than *Trichodesmium* in Fe-limited oceans (e.g., in western North Pacific Subtropical Gyre) [44], [45].

## 5 Conclusions

In summary, our study provides a deep insight into how *Trichodesmium* trichomes diurnally allocate their intracellular Fe to promote their growth. In addition to improving the Fe use efficiencies, dynamic Fe allocation is also involved in the intracellular O_2_ management, reducing the need for respiratory protection in producing low-O_2_ windows for N_2_ fixation. Our model framework could be further integrated with the microenvironment of *Trichodesmium* colonies to explore their in-depth physiological mechanisms regulating N_2_ fixation. It can also serve as an eco-physiological module in the biogeochemical model to promote the predictive ability in the context of oligotrophication of surface open ocean, especially under iron limitation.

## Author statement

YWL originated concept for the study. YWL and WL designed numerical model. WL coded the initial version of the model and performed numerical modeling. YWL and WL analyzed results and improved the numerical model. WL wrote the first draft of the manuscript; YWL and WL revised the manuscript.

## CRediT authorship contribution statement

**Weicheng Luo:** Methodology, Visualization, Validation, Writing – original draft, Writing – review & editing. **Ya-Wei Luo:** Conceptualization, Methodology, Validation, Writing – review & editing.

## Declaration of Competing Interest

The authors declare that they have no competing interests.
