# Supplementary material for: Diurnally dynamic iron allocation promotes N2 fixation in marine dominant diazotroph Trichodesmium
Source: Comput Struct Biotechnol J. 2023 Jul 6;21:3503–12. doi: 10.1016/j.csbj.2023.07.006 (PMC10362294; doi:10.1016/j.csbj.2023.07.006)
Supplement: Supplementary file 1 — Supplementary material [file mmc1.pdf]

Diurnally dynamic iron allocation promotes N_2 fixation in marine dominant diazotroph *Trichodesmium*

Weicheng Luo, Ya-Wei Luo

Supplementary Methods

1. Photosynthetic pathways

Linear photosynthetic electron transfer (LPET) and alternative electron transfer (AET) are simulated in our model (Fig. 1A). Because (Mehler reaction)-mediated AET is the dominant AET in *Trichodesmium* [1], other AET (e.g., the cyclic electron transfer around photosystem I, the AET mediated by midstream oxidase with photosystem II, and the AET from photosystem II to the respiratory terminal oxidase respiratory terminal oxidase) [2], are not considered.

Both LPET and AET generate a proton gradient of 12 H^+ per 8 photons [3]. By assuming that 3 ATP are produced by the thylakoid ATP synthase with a proton gradient of 14 H^+, for 4 electrons through PET, LPET produces 2.6 ATP, with 2 NADPH and 1 O_2, while AET only produces 2.6 ATP [2-4].

The total PET rate [V_{PET}, mol electron (mol C)$^{-1}$ s^{-1}] is regulated by light intensity (I, μmol m^{-2} s^{-1}) and the Fe quota in photosystems [Fe_{PS}, μmol Fe (mol C)$^{-1}$], and V_{PET} is inhibited by respiratory protection (RP) [V_{RP}, mol C (mol C)$^{-1}$ s^{-1}, described later] [5].

$$V_{PET} = v_{PET}^{max} \cdot \frac{Fe_{PS}}{Fe_{PS} + k_{Fe}^{PS}} \cdot (1 - e^{-\alpha_I \cdot I}) \cdot e^{-\beta \cdot V_{RP}}, \tag{S1}$$

where v_{PET}^{max} [mol electron (mol C)$^{-1}$ s^{-1}] is the maximal rate of PET, k_{Fe}^{PS} [μmol Fe (mol C)$^{-1}$] is the half-saturating coefficient of Fe_{PS} for PET, α_I (μmol^{-1} m^2 s) is the initial slope of PET versus light curve, and β [mol C (mol C)$^{-1}$ s] represents the degree of the inhibition effect from RP on PET. Note that in fixed-Fe and dynamic-Fe cases, the light intensity is diurnally variant with time, following the sine function during a 12-hour light period in our model [6].

To determine the rates of LPET and AET [V_{LPET} and V_{AET} , mol electron (mol C)$^{-1}$ s^{-1}], the fraction of photosynthetic electrons flowing into AET (f_{AET}, dimensionless) is introduced in our model, and f_{AET} is calculated in each time step to fulfill the intracellular immediate requirement of ATP and NADPH [7].

$$V_{LPET} = V_{PET} \cdot (1 - f_{AET}), \tag{S2}$$

$$V_{AET} = V_{PET} \cdot f_{AET}. \tag{S3}$$

The NADPH production rate [V_{NAPDH}, mol NADPH (mol C)$^{-1}$ s^{-1}] is:

$$V_{NAPDH} = V_{LPET} \cdot q_{LPET}^{NADPH}, \tag{S4}$$

where q_{LPET}^{NADPH} = 0.5 mol NADPH (mol electron)$^{-1}$ is the ratio of NADPH to electron in LPET [4].

The ATP production rate [V_{ATP}, mol ATP (mol C)$^{-1}$ s^{-1}] is:

$$V_{ATP} = V_{LPET} \cdot q_{LPET}^{ATP} + V_{AET} \cdot q_{AET}^{ATP}, \tag{S5}$$

where $q_{LPET}^{ATP} = q_{AET}^{ATP}$ = 0.65 mol ATP (mol electron)$^{-1}$ ratios of ATP to electron in LPET and AET [2-4].

The O_2 production rate [V_{O_2}, mol O_2 (mol C)$^{-1}$ s^{-1}] is:

$$V_{O_2} = V_{LPET} \cdot q_{LPET}^{O_2}, \tag{S6}$$

where $q_{LPET}^{O_2}$ = 0.25 mol O_2 (mol electron)$^{-1}$ is the ratio of O_2 to electron in LPET [3, 4].

2. N_2 fixation

N_2 fixation requires both ATP and NADPH [8, 9]. The maximal N_2 fixation rate [V_{NF}^{max}, mol N (mol C)$^{-1}$ s^{-1}] is calculated based on the assumption that produced ATP and NADPH of PET are completely consumed by N_2 fixation.

$$V_{NF}^{max} = \frac{V_{ATP}}{q_{NF}^{ATP}}, \tag{S7}$$

where q_{NF}^{ATP} = 9 mol ATP (mol N)$^{-1}$ is ATP:N ratio in N_2 fixation [8, 9].

The rate [V_{NF}, mol N (mol C)$^{-1}$ s^{-1}] is also regulated by the Fe quota in nitrogenase [Fe_{NF}, μmol Fe (mol C)$^{-1}$] and inhibited by intracellular O_2 [O_2, mol O_2 m^{-3}].

$$V_{NF} = V_{NF}^{max} \cdot \frac{Fe_{NF}}{Fe_{NF} + k_{Fe}^{NF}} \cdot (1 - \frac{O_2}{O_2 + k_{O_2}^{NF}}), \tag{S8}$$

where k_{Fe}^{NF} [μmol Fe (mol C)$^{-1}$] and $k_{O_2}^{NF}$ (mol O_2 m^{-3}) are half-saturating coefficients of Fe_{NF} and O_2 for N_2 fixation.

The NADPH and ATP consumption rates of N_2 fixation [V_{NADPH}^{NF} and V_{ATP}^{NF}, mol NADPH (mol C)$^{-1}$ s^{-1} and mol ATP (mol C)$^{-1}$ s^{-1}] are:

$$V_{NADPH}^{NF} = V_{NF} \cdot q_{NF}^{NADPH}, \tag{S9}$$

$$V_{ATP}^{NF} = V_{NF} \cdot q_{NF}^{ATP}. \tag{S10}$$

where q_{NF}^{NADPH} = 3 mol NADPH (mol N)$^{-1}$ is NADPH:N ratio in N_2 fixation [8, 9].

3. CO_2 concentrating mechanism and carbon fixation

The energy requirement of the CO_2 concentrating mechanism (CCM) [q_{CCM}^{ATP} = 0.8 mol ATP (mol C)$^{-1}$] is calculated based on the fraction of leakage (50%) of inorganic carbon (C_i), the fraction of HCO_3^- in C_i (80%), and the cost for per HCO_3^- transportation [0.5 mol ATP (mol C)$^{-1}$] [10, 11].

Energy consumption rate of CCM [V_{ATP}^{CCM}, mol ATP (mol C)$^{-1}$ s^{-1}] is:

$$V_{ATP}^{CCM} = V_{CF} \cdot q_{CCM}^{ATP}, \tag{S11}$$

where V_{CF} [mol C (mol C)$^{-1}$ s^{-1}] is carbon fixation rate.

Carbon fixation also requires both NADPH and ATP [12], and consumption rates [V_{NADPH}^{CF} and V_{ATP}^{CF}, mol NADPH (mol C)$^{-1}$ s^{-1} and mol ATP (mol C)$^{-1}$ s^{-1}] are:

$$V_{NADPH}^{CF} = V_{CF} \cdot q_{CF}^{NADPH}, \tag{S12}$$

$$V_{ATP}^{CF} = V_{CF} \cdot q_{CF}^{ATP}. \tag{S13}$$

V_{CF} is solved at each time step with f_{AET}, based on the consumption that total NADPH and ATP production by PET are immediately and fully utilized by intracellular process:

$$V_{NADPH} = V_{NADPH}^{CF} + V_{NADPH}^{NF}, \tag{S14}$$

$$V_{ATP} = (V_{ATP}^{CCM} + V_{ATP}^{CF} + V_{ATP}^{NF}) \cdot (1 + \gamma_{MT}), \tag{S15}$$

where γ_{MT} (dimensionless) represents the ratio of ATP consumption by maintenance to other processes.

The carbon skeleton production rate [V_{CS}, mol C (mol C)$^{-1}$ s^{-1}] is stimulated by carbohydrate [CH_2O, mol C (mol C)$^{-1}$] and downregulated by its own accumulation [CS, mol C (mol C)$^{-1}$]:

$$V_{CS} = v_{CS}^{max} \cdot \frac{CH_2O}{CH_2O + k_{CH_2O}^{CS}} \cdot \frac{CS_{max} - CS}{CS_{max}}, \tag{S16}$$

where v_{CS}^{max} [mol C (mol C)$^{-1}$ s^{-1}] is the maximal production rate of the carbon skeleton, $k_{CH_2O}^{CS}$ [mol C (mol C)$^{-1}$] is the half-saturation constant of carbohydrates for carbon skeleton production, and CS_{max} [mol C (mol C)$^{-1}$] is the maximum CS storage.

4. Respiratory protection

Respiratory protection (RP) rate is regulated by the demand for N_2 fixation and intracellular O_2 [13, 14, 7]:

$$V_{RP} = v_{RP}^{max} \cdot (1 - e^{-\alpha_I \cdot I}) \cdot \frac{CS}{CS + k_{CS}} \cdot \left(\frac{N_{max} - N}{N_{max}}\right) \cdot \frac{O_2}{O_2 + k_{O_2}^{NF}}, \tag{S17}$$

where v_{RP}^{max} [mol C (mol C)$^{-1}$ s^{-1}] is the maximal respiratory protection rate, k_{CS} [mol C (mol C)$^{-1}$] is the half-saturating coefficient of the carbon skeleton for respiratory protection, and N_{max} [mol N (mol C)$^{-1}$] is the maximal N storage.

The O_2 consumption by RP [$V_{O_2}^{RP}$, mol O_2 (mol C)$^{-1}$ s^{-1}] is:

$$V_{O_2}^{RP} = V_{RP} \cdot q_{C}^{O_2}, \tag{S18}$$

where $q_{C}^{O_2}$ [mol O_2 (mol C)$^{-1}$] is the ratio of O_2 to carbon in carbohydrate respiration.

5. O_2 diffusion

The rate of O_2 diffusion (T_{O_2}, mol O_2 m^{-3} s^{-1}) between intracellular cytoplasm and ambient environment is parameterized by adopting the scheme of [15]:

$$T_{O_2} = \frac{-2 \cdot \pi \cdot d_{O_2} \cdot L}{V} \cdot \left\{ \frac{1}{\varepsilon} \cdot \ln\left(\frac{R}{R + L_g}\right) - \ln\left(\frac{R + L_g + L_b}{R + L_g}\right) \right\}^{-1} \cdot (O_2^E - O_2), \quad \text{(S19)}$$

where d_{O_2} (m^2 s^{-1}) is the O_2 diffusion coefficient in seawater, ε (dimensionless) is the ratio of the O_2 diffusion coefficient of the cell membrane relative to d_{O_2}, L (m) and V (m^3) are the length and the volume of the trichome, R (m) is the radius of the cytoplasm, L_g (m) is the thickness of the cell membrane, L_b (m) is the thickness of the boundary layer, O_2^E is the ambient far-field O_2 concentration.

6. Intracellular Fe pools and translocation

Trichodesmium can uptake more Fe than that required by its metabolism (called 'luxury uptake') especially in high-Fe environments, and the excess Fe is stored for surviving in low Fe environments [16, 17]. Therefore, the total intracellular Fe quota [Fe, µmol Fe (mol C)$^{-1}$] consists of Fe in metabolism and storage [Fe_M and Fe_{ST}, µmol Fe (mol C)$^{-1}$], calculated based on the threshold of Fe [Fe_{TH}, µmol Fe (mol C)$^{-1}$] [18].

$$Fe_M = Fe, \quad \text{when } Fe \leq Fe_{TH}, \quad \text{(S20)}$$

$$Fe_M = Fe_{TH} + (1 - f_{ST}) \cdot (Fe - Fe_{TH}), \text{ when } Fe > Fe_{TH}, \quad \text{(S21)}$$

where f_{ST} (dimensionless) is the fraction of luxury Fe uptake.

Fe allocations are among Fe_M, including Fe in photosystems, active nitrogenase, inactivated nitrogenase, maintenance and buffer [Fe_{PS}, Fe_{NF}, Fe_{NF}^{NA}, Fe_{MT} and Fe_{BF}, µmol Fe (mol C)$^{-1}$] (Fig. 1B). Fe in maintenance is set diurnally constant at 10% of Fe_M [18]. Fe used in the photosystems and nitrogenase is from the buffer pool [14].

The synthesis rate of photosystems [T_{PS}^{BF}, µmol Fe (mol C)$^{-1}$ s^{-1}] is stimulated by light intensity and is gradually saturated with Fe_{PS}:

$$T_{PS}^{BF} = TF_{PS_{max}}^{BF} \cdot (1 - e^{-\alpha_I \cdot I}) \cdot (1 - \frac{Fe_{PS}}{Fe_{PS} + k_{Fe_{PS}}^{PS_{syn}}}), \quad \text{(S22)}$$

where $T_{PS_{max}}^{BF}$ [µmol Fe (mol C)$^{-1}$ s^{-1}] is the maximal synthesis rate of photosystems, $k_{Fe_{PS}}^{PS_{syn}}$ [µmol Fe (mol C)$^{-1}$] is the half-saturating coefficients of Fe_{PS} for the synthesis of photosystems.

The decomposition rate of photosystems [T_{BF}^{PS}, μmol Fe (mol C)$^{-1}$ s^{-1}] is stimulated by Fe_{PS} but inhibited by respiratory protection [5]:

$$T_{BF}^{PS} = T_{BF_{max}}^{PS} \cdot \frac{Fe_{PS}}{Fe_{PS} + k_{Fe_{PS}}^{PS_{dec}}} \cdot e^{-\beta \cdot V_{RP}}, \tag{S23}$$

where $T_{BF_{max}}^{PS}$ [μmol Fe (mol C)$^{-1}$ s^{-1}] is the maximal decomposition rate of photosystems, $k_{Fe_{PS}}^{PS_{dec}}$ [μmol Fe (mol C)$^{-1}$] is the half-saturating coefficient of Fe_{PS} for the decomposition of photosystems. Fe released from decomposed photosystems returns to buffer pool [14].

For nitrogenase, intracellular requirement for N_2 fixation and Fe_{BF} regulate its synthesis rate [T_{NF}^{BF}, μmol Fe (mol C)$^{-1}$ s^{-1}]:

$$T_{NF}^{BF} = T_{NF_{max}}^{BF} \cdot (1 - e^{-\alpha_I \cdot I}) \cdot \frac{CS}{CS + k_{CS}} \cdot (\frac{N_{max} - N}{N_{max}}) \cdot \frac{Fe_{BF}}{Fe_{BF} + k_{Fe_{BF}}^{NF_{syn}}}, \tag{S24}$$

where $T_{NF_{max}}^{BF}$ [μmol Fe (mol C)$^{-1}$ s^{-1}] is the maximal nitrogenase synthesis rate, k_{CS} [mol C (mol C)$^{-1}$] and $k_{Fe_{BF}}^{NF_{syn}}$ [μmol Fe (mol C)$^{-1}$] are half-saturating coefficients of the carbon skeleton and Fe_{BF} for the synthesis of nitrogenase, respectively.

The decomposition of nitrogenase seems to occur at night [19, 20], and therefore it is not considered during the light period in our model. Notably, nitrogenase is inhibited upon exposure to O_2, flowing into the pool of inactivated nitrogenase [5] at the rate [T_{NF}^{NA}, μmol Fe (mol C)$^{-1}$ s^{-1}]:

$$T_{NF}^{NA} = T_{NF_{max}}^{NA} \cdot \frac{Fe_{NF}}{Fe_{NF} + k_{Fe}^{NF}} \cdot \frac{O_2}{O_2 + k_{O_2}^{NF}}, \tag{S25}$$

where $T_{NF_{max}}^{NA}$ [μmol Fe (mol C)$^{-1}$ s^{-1}] is the maximal inactivation rate of nitrogenase.

Note that, in the fixed-Fe model case, photosystems and total nitrogenase are set diurnally constant, with zero synthesis or decomposition rates, but the inactivation of nitrogenase is still taken into consideration.

7. Integration of state variables during the daytime

The diurnal change rates (basically normalized to carbon biomass) of CH_2O, CS, N, intracellular O_2 and Fe are represented in ordinary differential equations (ODEs). Note that O_2 is in a unit volumetric concentration (mol O_2 m^{-3}):

$$\frac{dCH_2O}{dt} = V_{CF} - V_{CS} - V_{RP}, \tag{S26}$$

$$\frac{dCS}{dt} = V_{CS}, \tag{S27}$$

$$\frac{dN}{dt} = V_{NF}, \quad (S28)$$

$$\frac{dO_2}{dt} = (V_{O_2} - V_{O_2}^{RP}) \cdot Q_C + T_{O_2}, \quad (S29)$$

$$\frac{dFe_{PS}}{dt} = T_{PS}^{BF} - T_{BF}^{PS}, \quad (S30)$$

$$\frac{dFe_{NF}}{dt} = T_{NF}^{BF} - T_{NF}^{NA}, \quad (S31)$$

$$\frac{dFe_{NF}^{NA}}{dt} = T_{NF}^{NA}, \quad (S32)$$

$$\frac{dFe_{BF}}{dt} = T_{BF}^{PSI} - T_{PSI}^{BF} \cdot + T_{NF}^{BF}, \quad (S33)$$

where Q_C = 18333 mol C m^{-3} is the cellular carbon biomass quota [21]. ODEs are run over a12-hour light period with ode15s integrator of MATLAB [22].

8. Biosynthesis and growth rate

Trichodesmium might store newly fixed C and N during the daytime and assimilate them into biomass, mainly during the dark period [23]. Therefore, for simplification, no biomass is synthesized during the light period in the model. Instead, the model calculates the amount of biomass [*Bio*, mol C (mol C)$^{-1}$] that can be synthesized using the carbohydrates, carbon skeletons and fixed N at the end of the light period. *Bio* is the smaller of N-based (Bio_N) and C-based biomass (Bio_C), with Bio_N calculated by dividing fixed N to the molar N:C (0.159) [24]. Bio_C is calculated from the carbohydrates and carbon skeleton considering mass and energy balance. The energy needed for biosynthesis is from the respiration of carbohydrates ($CH_2O_{BIO}^{RESP}$):

$$Bio_C \cdot q_{BIO}^{ATP} \cdot (1 + \gamma_{MT}) = CH_2O_{BIO}^{RESP} \cdot q_{RESP}^{ATP}, \quad (S34)$$

where q_{BIO}^{ATP} = 2 mol ATP (mol C)$^{-1}$ is the ATP requirement rate by biosynthesis [13], and q_{RESP}^{ATP} = 5 mol ATP (mol C)$^{-1}$ is the ATP production rate from respiring carbohydrates [25]. Meanwhile, the non-respired carbohydrates and all the carbon skeletons are involved in biosynthesis:

$$Bio_C = CH_2O - CH_2O_{BIO}^{RESP} + CS. \quad (S35)$$

Bio_C then can be solved from the above two equations. Note that the carbohydrate respiration calculated in this step is counted in the daily integrated respiration as the ordinary respiration.

Noting that all the rates have been normalized to carbon biomass, *Bio* is therefore the relative increase in biomass over one day. The growth rate (*G*) is then the natural log of (1 + *Bio*) divided by 1 day.

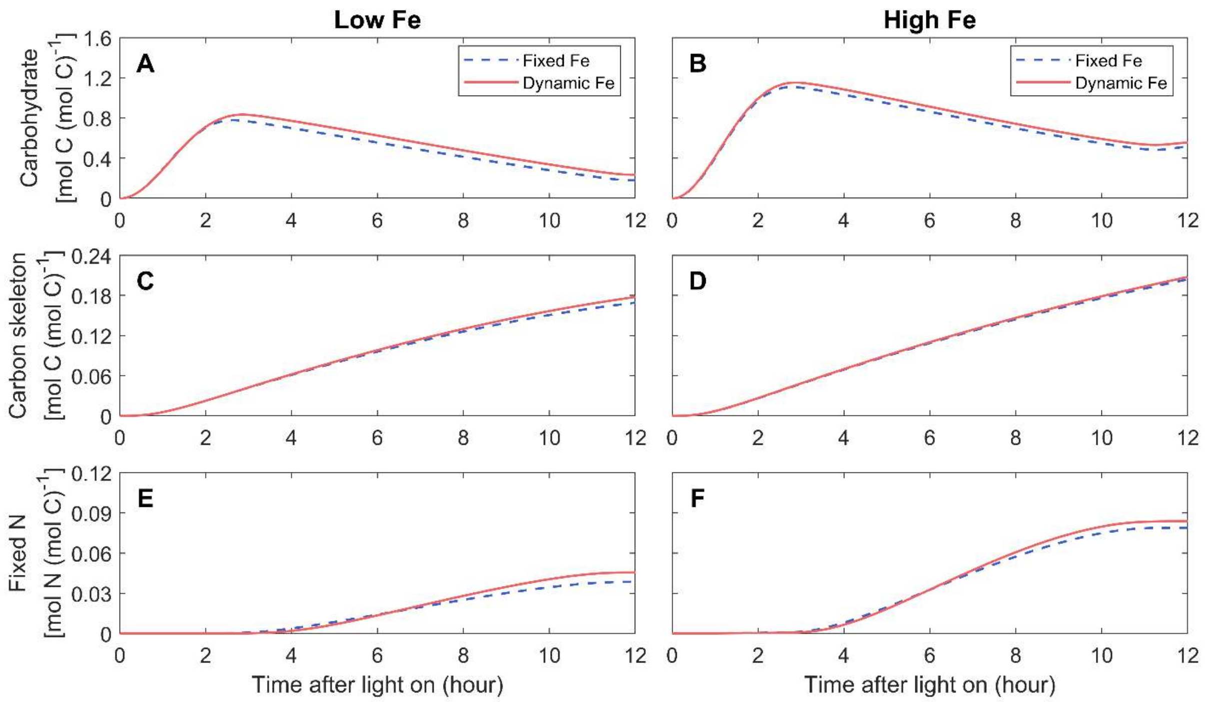

Fig. S1. Simulated carbohydrate, carbon skeleton and fixed N during the light period. The model is simulated with diurnally fixed or dynamic Fe allocations under low-Fe (40 pM) (**A**, **C** and **E**) and high-Fe (1250 pM) (**B**, **D** and **F**) conditions.

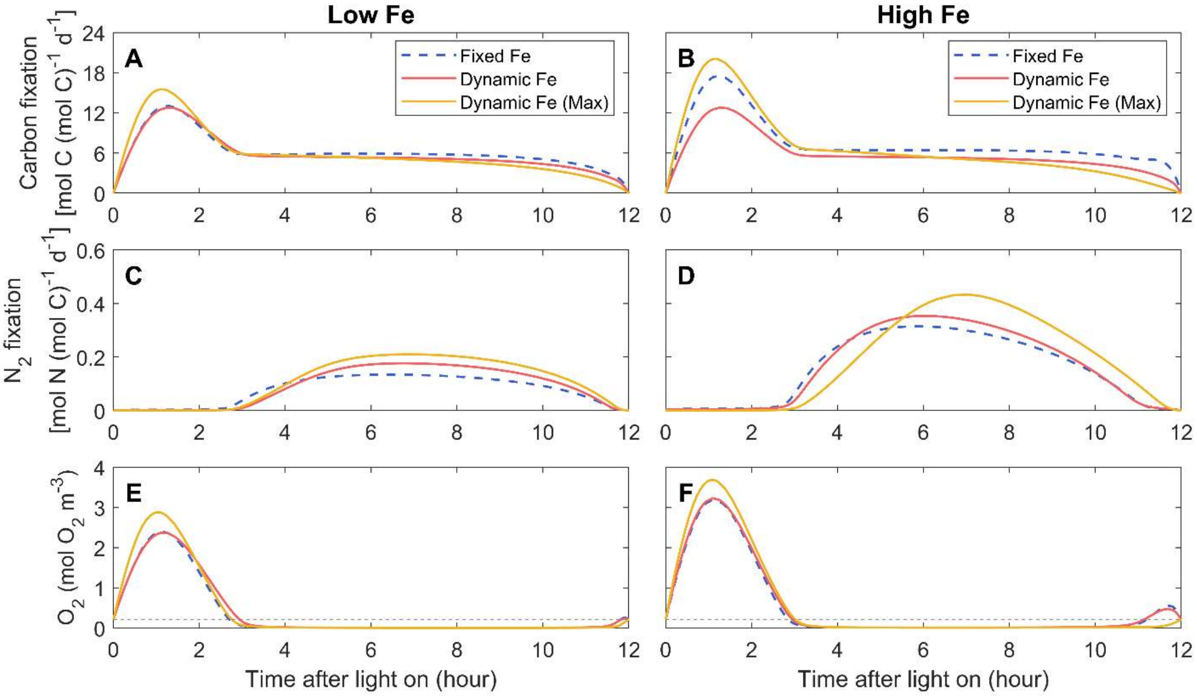

Fig. S2. Results of model experiments with maximized initial photosystem Fe. The dynamic-Fe (Max) case is same as the standard dynamic-Fe case, except that the initial Fe in photosystems is maximized to 90% of total metabolic Fe. The experiments are conducted under low-Fe (40 pM) (**A**, **C** and **E**) and high-Fe (1250 pM) (**B**, **D** and **F**) conditions. For comparison, the results of standard fixed-Fe and dynamic-Fe cases are also shown.

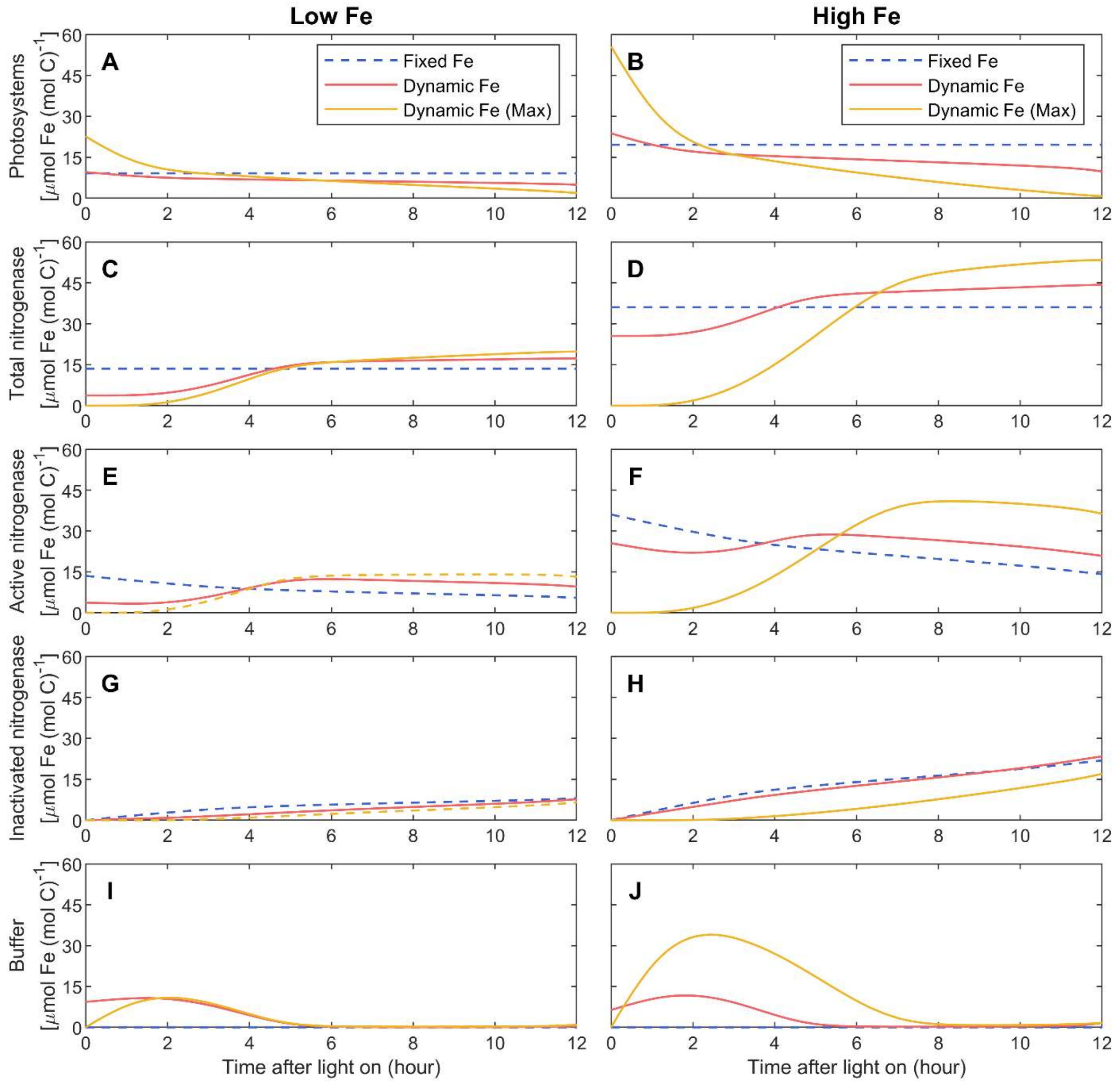

Fig. S3. Results of model experiments with maximized initial photosystem Fe. See the caption of Figure S2 for the experiment setup.

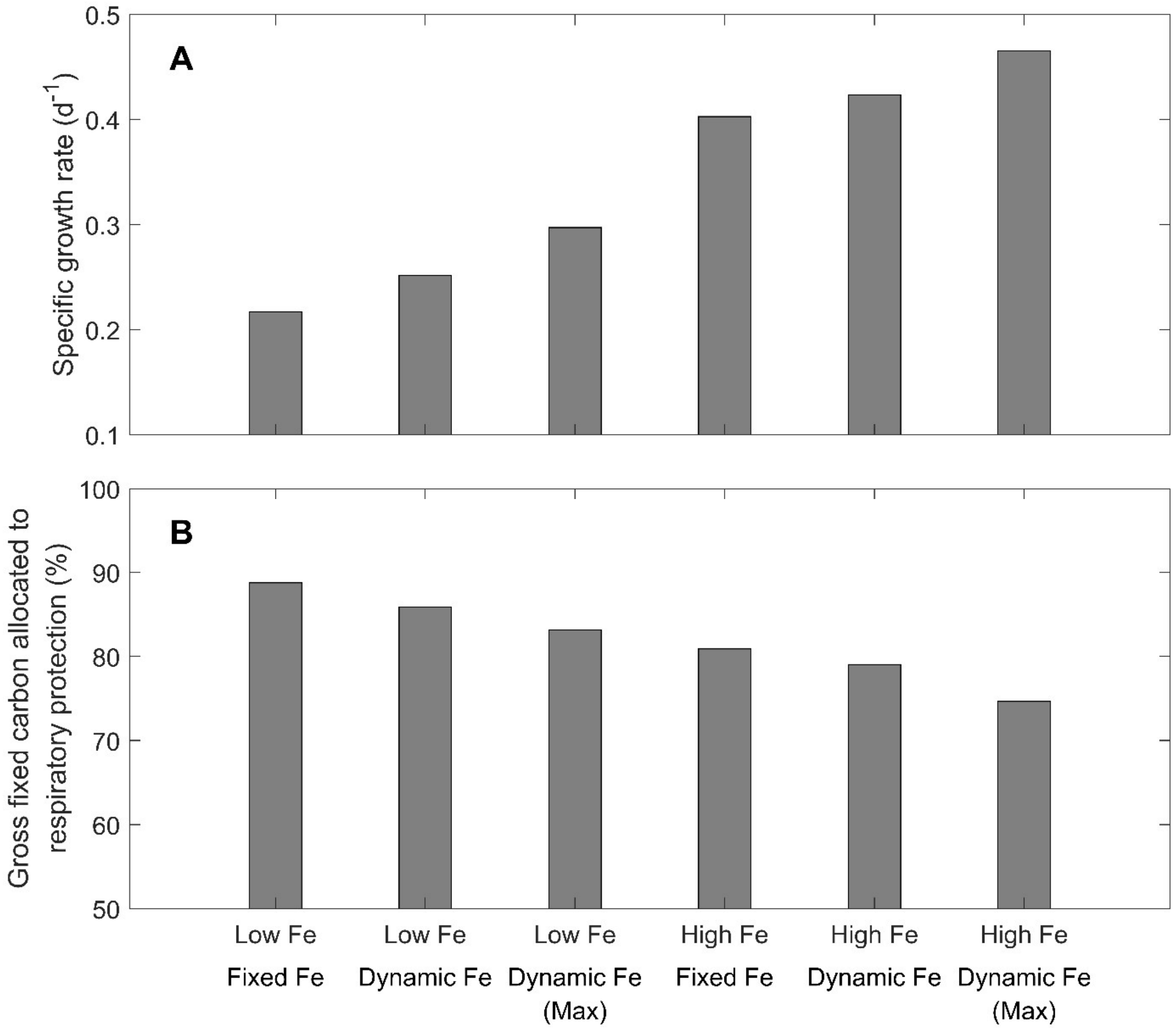

Fig. S4. Results of model experiments with maximized initial photosystem Fe. See the caption of Figure S2 for the experiment setup.

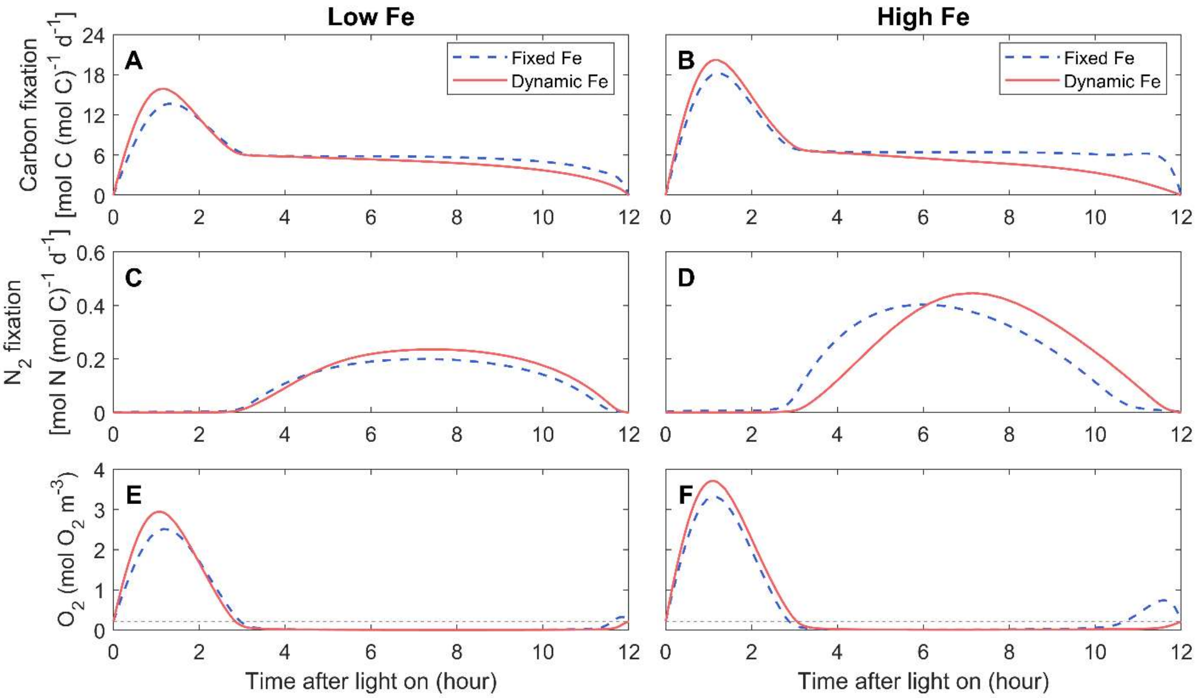

Fig. S5. Results of model experiments with decomposing inactivated nitrogenase and maximized initial photosystem Fe. The inactivated nitrogenase is immediately decomposed and its Fe is released to the buffer pool. The experiments are conducted with diurnally fixed or diurnally dynamic Fe allocations, with the initial Fe in photosystems being maximized to 90% of total metabolic Fe in the dynamic-Fe experiments. The experiments are conducted under low-Fe (40 pM) (**A**, **C** and **E**) and high-Fe (1250 pM) (**B**, **D** and **F**) conditions.

Table S1. Optimized parameters

Symbol	Unit	Definition	Value			
			Low Fe (40 pM)		High Fe (1250 pM)	
			Fixed-Fe case	Dynamic-Fe case	Fixed-Fe case	Dynamic-Fe case
v_{RP}^{max}	mol C (mol C)$^{-1}$ s^{-1}	Maximal respiratory protection rate	1.4×10^{-3}	1.1×10^{-3}	1.3×10^{-3}	1.2×10^{-3}
$T_{PS_{max}}^{BF}$	μmol Fe (mol C)$^{-1}$ s^{-1}	Maximal synthesis rate of photosystems	0*	2.0×10^{-6}	0*	1.5×10^{-6}
$T_{BF_{max}}^{PS}$	μmol Fe (mol C)$^{-1}$ s^{-1}	Maximal decomposition rate of photosystems	0*	1.4×10^{-3}	0*	2.6×10^{-3}
$T_{NF_{max}}^{BF}$	μmol Fe (mol C)$^{-1}$ s^{-1}	Maximal synthesis rate of nitrogenase	0*	2.4×10^{-2}	0*	2.8×10^{-2}
$f_{Fe_0}^{PS}$	dimensionless	Fraction of metabolic Fe in initial photosystems	36%	38%†	32%	38%†
$f_{Fe_0}^{NF}$	dimensionless	Fraction of metabolic Fe in initial nitrogenase	54%	15%†	58%	41%†

* Note that synthesis rates of photosystems and nitrogenase, and decomposition rate of photosystems are set zero in the fixed-Fe case.

† Note that $f_{Fe_0}^{PS}$ and $f_{Fe_0}^{NF}$ in the dynamic-Fe case are set the same as observational data from [19].

Table S2. Fixed model parameters

Symbol	Unit	Definition	Value	Source or Note
v_{PET}^{max}	mol electron (mol C)$^{-1}$ s^{-1}	Maximal PET rate	6.6×10^{-3}	This study*
k_{Fe}^{PS}	μmol Fe (mol C)$^{-1}$	Half-saturating coefficient of Fe in photosystems for PET rate	10	This study*
v_{CS}^{max}	mol C (mol C)$^{-1}$ s^{-1}	Maximal production rate of CS	8.6×10^{-6}	This study*
k_{CS}	mol C (mol C)$^{-1}$	Half-saturating coefficient of CS for RP	0.4	This study*
$k_{CH_2O}^{CS}$	mol C (mol C)$^{-1}$	Half-saturating coefficient of CH_2O for CS production	0.4	This study*
$k_{Fe_{PS}}^{PS_{syn}}$	μmol Fe (mol C)$^{-1}$	Half-saturating coefficient of Fe_{PS} for the synthesis of photosystems	1.0	This study*
$k_{Fe_{PS}}^{PS_{dec}}$	μmol Fe (mol C)$^{-1}$	Half-saturating coefficient of Fe_{PS} for the decomposition of photosystems	25	This study*
$k_{Fe_{BF}}^{NF_{syn}}$	μmol Fe (mol C)$^{-1}$	Half-saturating coefficient of Fe_{BF} for the synthesis of nitrogenase	5.0	This study*
$T_{NF_{max}}^{NA}$	μmol Fe (mol C)$^{-1}$ s^{-1}	Maximal inactivation rate of nitrogenase	3.3×10^{-3}	This study*
N_{max}	mol N (mol C)$^{-1}$	Maximal fixed storage	0.159	This study†
CS_{max}	mol C (mol C)$^{-1}$	Maximal CS storage	1	This study‡
α_I	μmol^{-1} m^2 s	Initial slope of P versus I curve	0.01	[13]
β	(mol C)$^{-1}$ mol C s	Parameter of inhibition effect of respiration on PET	2×10^4	[7]
$k_{O_2}^{NF}$	mol O_2 m^{-3}	Half-saturating coefficient of O_2 for N_2 fixation	0.01	[7]
ε	dimensionless	Relative diffusivity of cell membrane	10^{-4}	[7]
d_{O_2}	m^2 s^{-1}	O_2 diffusion coefficient at 34 PSU and 25 °C	2.26×10^{-9}	[26]
k_{Fe}^{NF}	μmol Fe (mol C)$^{-1}$	Half-saturating coefficient of Fe in nitrogenase for N_2 fixation	91	[18]
γ_{MT}	dimensionless	Ratio of the energy consumed by maintenance to other process	10%	[18]
Fe_{TH}	μmol Fe (mol C)$^{-1}$	Threshold of intracellular metabolic Fe quota	24.4	[18]
f_{ST}	dimensionless	Fraction of luxury Fe uptake	0.90	[18]
		Boundary conditions		
I_{max}	μmol m^{-2} s^{-1}	Maximal light intensity in fixed-Fe and dynamic-Fe cases under diurnally changing light intensity	160	
O_2^E	mol O_2 m^{-3}	Extracellular far-field O_2	0.213	
		Elemental or energy stoichiometries of metabolic activities		
q_{LPET}^{NADPH}	mol NADPH (mol electron)$^{-1}$	NADPH/electron ratio of LPET	0.5	[4]
q_{LPET}^{ATP}	mol ATP (mol electron)$^{-1}$	ATP/electron ratio of LPET	0.65	[3]
$q_{LPET}^{O_2}$	mol O_2 (mol electron)$^{-1}$	O_2/electron ratio of LPET	0.25	[4]
q_{AET}^{ATP}	mol ATP (mol electron)$^{-1}$	ATP/electron ratio of MR-AET	0.65	[3]
q_{NF}^{NADPH}	mol NADPH (mol N)$^{-1}$	NADPH/N ratio of N_2 fixation	3	[8, 9]
q_{NF}^{ATP}	mol ATP (mol N)$^{-1}$	ATP/N ratio of N_2 fixation	9	[8, 9]
q_{CF}^{NADPH}	mol NADPH (mol C)$^{-1}$	NADPH/C ratio of C fixation	2	[12]
q_{CF}^{ATP}	mol ATP (mol C)$^{-1}$	ATP/C ratio of C fixation	3	[12]
q_{CCM}^{ATP}	mol ATP (mol C)$^{-1}$	ATP/C ratio of CCM	0.8	[11]
q_{BIO}^{ATP}	mol ATP (mol C)$^{-1}$	ATP/C ratio of biosynthesis	2	[13]

q_{RESP}^{ATP}	mol ATP (mol C)$^{-1}$	ATP/C ratio of respiration	5	[25]
$q_{C}^{O_2}$	mol O_2 (mol C)$^{-1}$	O_2/C ratio of respiration	1	[25]
Q_C	mol C m^{-3}	Cellular carbon biomass concentration	18333	[21]
		Morphological parameters of Trichodesmium		
L	m	Length of the total trichome	554×10^{-6}	[27]
R	m	Radius of the cytoplasm	4.80×10^{-6}	[27]
L_g	m	Thickness of cell membrane layer	0.076	[27]

* Estimated based on model experiments under constant light intensity.

† By multiplying the initial C biomass with the molar N:C (0.159) of *Trichodesmium* [24].

‡ CS_{max} is set to be the same as the initial C biomass.

Table S3. Intermediate process variables

Symbol	Unit	Definition
V_{PET}	mol electron (mol C)$^{-1}$ s^{-1}	PET rate
V_{LPET}	mol electron (mol C)$^{-1}$ s^{-1}	LPET rate
V_{AET}	mol electron (mol C)$^{-1}$ s^{-1}	AET rate
f_{AET}	dimensionless	Fraction of electrons in PET to AET
V_{NAPDH}	mol NADPH (mol C)$^{-1}$ s^{-1}	NADPH production rate
V_{ATP}	mol ATP (mol C)$^{-1}$ s^{-1}	ATP production rate
V_{O_2}	mol O_2 (mol C)$^{-1}$ s^{-1}	O_2 production rate
V_{NF}^{max}	mol N (mol C)$^{-1}$ s^{-1}	Maximal N_2 fixation rate
V_{NF}	mol N (mol C)$^{-1}$ s^{-1}	N_2 fixation rate
V_{NADPH}^{NF}	mol NADPH (mol C)$^{-1}$ s^{-1}	NADPH consumption rate of N_2 fixation
V_{ATP}^{NF}	mol ATP (mol C)$^{-1}$ s^{-1}	ATP consumption rate of N_2 fixation
V_{ATP}^{CCM}	mol ATP (mol C)$^{-1}$ s^{-1}	ATP consumption rate of CCM
V_{NADPH}^{CF}	mol NADPH (mol C)$^{-1}$ s^{-1}	NADPH consumption rate of C fixation
V_{ATP}^{CF}	mol ATP (mol C)$^{-1}$ s^{-1}	ATP consumption rate of C fixation
V_{CS}	mol C (mol C)$^{-1}$ s^{-1}	Carbon skeleton production rate
V_{RP}	mol C (mol C)$^{-1}$ s^{-1}	Respiratory protection rate
$V_{O_2}^{RP}$	mol O_2 (mol C)$^{-1}$ s^{-1}	O_2 consumption rates by respiratory protection
T_{O_2}	mol O_2 m^{-3} s^{-1}	O_2 diffusion rate between cytoplasm and ambient environment
Fe_M	μmol Fe (mol C)$^{-1}$	Intracellular Fe quota in metabolism
Fe_{ST}	μmol Fe (mol C)$^{-1}$	Intracellular Fe quota in storage
T_{PS}^{BF}	μmol Fe (mol C)$^{-1}$ s^{-1}	Synthesis rate of photosystems
T_{BF}^{PS}	μmol Fe (mol C)$^{-1}$ s^{-1}	Decomposition rate of photosystems
T_{NF}^{BF}	μmol Fe (mol C)$^{-1}$ s^{-1}	Synthesis rate of nitrogenase
T_{NF}^{NA}	μmol Fe (mol C)$^{-1}$ s^{-1}	Inactivation rate of nitrogenase
Φ	dimensionless	Intracellular requirement of N_2 fixation
Bio	mol C (mol C)$^{-1}$	New synthesized biomass
Bio_N	mol C (mol C)$^{-1}$	New synthesized N-based biomass
Bio_C	mol C (mol C)$^{-1}$	New synthesized C-based biomass
$CH_2O_{BIO}^{RESP}$	mol C (mol C)$^{-1}$	Respired carbohydrates to fulfill the energy need for biosynthesis
G	d^{-1}	Specific growth rate

Table S4. State variables

Symbol	Unit	Definition
CH_2O	mol C (mol C)$^{-1}$	Carbohydrate
CS	mol C (mol C)$^{-1}$	Carbon skeleton
N	mol N (mol C)$^{-1}$	Fixed N
O_2	mol O_2 m^{-3}	Intracellular O_2
Fe_{PS}	μmol Fe (mol C)$^{-1}$	Fe in photosystems
Fe_{NF}	μmol Fe (mol C)$^{-1}$	Fe in active nitrogenase
Fe_{NF}^{NA}	μmol Fe (mol C)$^{-1}$	Fe in inactivated nitrogenase
Fe_{BF}	μmol Fe (mol C)$^{-1}$	Fe in buffer

Note: The initial values (t = 0) of CH_2O, CS and N are set to be 0, and initial O_2 concentration is the same as that of ambient O_2 (0.213 mol O_2 m^{-3}).

References

[1] Milligan AJ, Berman-Frank I, Gerchman Y, Dismukes GC, Falkowski PG. Light-dependent oxygen consumption in nitrogen-fixing cyanobacteria plays a key role in nitrogenase protection. J Phycol 2007;43(5):845-52.

[2] Behrenfeld MJ, Milligan AJ. Photophysiological expressions of iron stress in phytoplankton. Annu Rev Mar Sci 2013;5:217-46.

[3] Geider RJ, Moore CM, Ross ON. The role of cost–benefit analysis in models of phytoplankton growth and acclimation. Plant Ecol Div 2009;2(2):165-78.

[4] Allen JF. Cyclic, pseudocyclic and noncyclic photophosphorylation: New links in the chain. Trends Plant Sci 2003;8(1):15-9.

[5] Berman-Frank I, Lundgren P, Chen YB, Kupper H, Kolber Z, Bergman B, et al. Segregation of nitrogen fixation and oxygenic photosynthesis in the marine cyanobacterium *Trichodesmium*. Science 2001;294(5546):1534-7.

[6] Reimers AM, Knoop H, Bockmayr A, Steuer R. Cellular trade-offs and optimal resource allocation during cyanobacterial diurnal growth. Proc Natl Acad Sci USA 2017;114(31):E6457-E65.

[7] Luo W, Inomura K, Zhang H, Luo Y-W. N_2 Fixation in *Trichodesmium* Does Not Require Spatial Segregation from Photosynthesis. mSystems 2022;7(4):e00538-22.

[8] Flores E, Herrero A. Assimilatory nitrogen metabolism and its regulation. In: Bryant DA, editor The Molecular Biology of Cyanobacteria. Dordrecht: Kluwer Academic Publishers; 1994, p. 487-517.

[9] Flores E, Frías JE, Rubio LM, Herrero A. Photosynthetic nitrate assimilation in cyanobacteria. Photosynth Res 2005;83(2):117-33.

[10] Eichner M, Thoms S, Kranz SA, Rost B. Cellular inorganic carbon fluxes in *Trichodesmium*: A combined approach using measurements and modelling. J Exp Bot 2015;66(3):749-59.

[11] Raven JA, Beardall J, Giordano M. Energy costs of carbon dioxide concentrating mechanisms in aquatic organisms. Photosynth Res 2014;121(2-3):111-24.

[12] Baker NR, Harbinson J, Kramer DM. Determining the limitations and regulation of photosynthetic energy transduction in leaves. Plant Cell Environ 2007;30(9):1107-25.

[13] Inomura K, Wilson ST, Deutsch C. Mechanistic model for the coexistence of nitrogen fixation and photosynthesis in marine *Trichodesmium*. mSystems 2019;4(4):e00210-19.

[14] Inomura K, Deutsch C, Wilson ST, Masuda T, Lawrenz E, Lenka B, et al. Quantifying oxygen management and temperature and light dependencies of nitrogen fixation by *Crocosphaera watsonii*. mSphere 2019;4(6):e00531-19.

[15] Staal M, Meysman FJ, Stal LJ. Temperature excludes N_2-fixing heterocystous cyanobacteria in the tropical oceans. Nature 2003;425(6957):504-7.

[16] Kustka AB, Sañudo-Wilhelmy SA, Carpenter EJ, Capone D, Burns J, Sunda WG. Iron requirements for dinitrogen- and ammonium-supported growth in cultures of *Trichodesmium* (IMS 101): Comparison with nitrogen fixation rates and iron: carbon ratios of field populations. Limnol Oceanogr 2003;48:1869–84.

[17] Berman-Frank I, Cullen JT, Shaked Y, Sherrell RM, Falkowski PG. Iron availability, cellular iron quotas, and nitrogen fixation in *Trichodesmium*. Limnol Oceanogr 2001;46(6):1249-60.

[18] Luo YW, Shi D, Kranz SA, Hopkinson BM, Hong H, Shen R, et al. Reduced nitrogenase efficiency dominates response of the globally important nitrogen fixer *Trichodesmium* to ocean acidification. Nat Commun 2019;10(1):1521.

[19] Shi D, Kranz SA, Kim JM, Morel FM. Ocean acidification slows nitrogen fixation and growth in the dominant diazotroph *Trichodesmium* under low-iron conditions. Proc Natl Acad Sci U S A 2012;109(45):E3094-100.

[20] Capone DG, O'Neil JM, Zehr JP, Carpenter EJ. Basis for Diel Variation in Nitrogenase Activity in the Marine Planktonic Cyanobacterium *Trichodesmium thiebautii*. Applied and Environmental Microbiology 1990;56(11):3532-6.

[21] Bratbak G, Dundas I. Bacterial dry matter content and biomass estimations. Appl Environ Microbiol 1984;48(4):755-7.

[22] Shampine LF, Reichelt MW. The MATLAB ODE Suite. SIAM J Sci Comput 1997;18:1-22.

[23] Finzi-Hart JA, Pett-Ridge J, Weber PK, Popa R, Fallon SJ, Gunderson T, et al. Fixation and fate of C and N in the cyanobacterium *Trichodesmium* using nanometer-scale secondary ion mass spectrometry. Proc Natl Acad Sci USA 2009;106(15):6345-50.

[24] LaRoche J, Breitbarth E. Importance of the diazotrophs as a source of new nitrogen in the ocean. J Sea Res 2005;53(1-2):67-91.

[25] Mitchell P. Aspects of the chemiosmotic hypothesis. Biochem J 1970;116(4):5-6.

[26] Benson BB, Krause D. The concentration and isotopic fractionation of oxygen dissolved in freshwater and seawater in equilibrium with the atmosphere. Limnol Oceanogr 1984;29(3):620-32.

[27] Carpenter EJ, Oneil JM, Dawson R, Capone DG, Siddiqui PJA, Roenneberg T, et al. The tropical diazotrophic phytoplankter *Trichodesmium*: Biological characteristics of two common species. Mar Ecol Prog Ser 1993;95(3):295-304.
